# The quantitative trait locus *GWY10* controls rice grain width and yield

**DOI:** 10.1093/plphys/kiae456

**Published:** 2024-08-29

**Authors:** Waseem Abbas, Qi Sun, Yana Cui, Abdullah Shalmani, Pengkun Xu, Yawei Fan, Dejian Zhang, Meng-en Wu, Xingxing Li, Yibo Li

**Affiliations:** National Key Laboratory of Crop Genetic Improvement and National Centre of Plant Gene Research (Wuhan), Huazhong Agricultural University, Wuhan 430070, China; Hubei Hongshan Laboratory, Wuhan 430070, China; National Key Laboratory of Crop Genetic Improvement and National Centre of Plant Gene Research (Wuhan), Huazhong Agricultural University, Wuhan 430070, China; Hubei Hongshan Laboratory, Wuhan 430070, China; National Key Laboratory of Crop Genetic Improvement and National Centre of Plant Gene Research (Wuhan), Huazhong Agricultural University, Wuhan 430070, China; Hubei Hongshan Laboratory, Wuhan 430070, China; National Key Laboratory of Crop Genetic Improvement and National Centre of Plant Gene Research (Wuhan), Huazhong Agricultural University, Wuhan 430070, China; Hubei Hongshan Laboratory, Wuhan 430070, China; National Key Laboratory of Crop Genetic Improvement and National Centre of Plant Gene Research (Wuhan), Huazhong Agricultural University, Wuhan 430070, China; Hubei Hongshan Laboratory, Wuhan 430070, China; National Key Laboratory of Crop Genetic Improvement and National Centre of Plant Gene Research (Wuhan), Huazhong Agricultural University, Wuhan 430070, China; Hubei Hongshan Laboratory, Wuhan 430070, China; National Key Laboratory of Crop Genetic Improvement and National Centre of Plant Gene Research (Wuhan), Huazhong Agricultural University, Wuhan 430070, China; Hubei Hongshan Laboratory, Wuhan 430070, China; National Key Laboratory of Crop Genetic Improvement and National Centre of Plant Gene Research (Wuhan), Huazhong Agricultural University, Wuhan 430070, China; Hubei Hongshan Laboratory, Wuhan 430070, China; National Key Laboratory of Crop Genetic Improvement and National Centre of Plant Gene Research (Wuhan), Huazhong Agricultural University, Wuhan 430070, China; Hubei Hongshan Laboratory, Wuhan 430070, China; National Key Laboratory of Crop Genetic Improvement and National Centre of Plant Gene Research (Wuhan), Huazhong Agricultural University, Wuhan 430070, China; Hubei Hongshan Laboratory, Wuhan 430070, China

## Abstract

A rice grain width and yield quantitative trait locus increases grain yield in near-isogenic lines and gene-edited lines and improves grain appearance and milling quality.

Dear Editor,

Grain size, as a complex and multigenic trait, is the key determinant of rice (*Oryza sativa* L.) grain yield and quality, affecting grain number and quality and limiting the application of many grain size quantitative trait genes (Li et al. 2019; Sreenivasulu et al. 2022). Although multiple grain size genes have been cloned, their regulatory relationships between upstream and downstream components are still unknown (Ren et al. 2023). Conserved actin and cyclin-dependent kinases (CDKs) play crucial roles in plant cell division (Wang and Mao 2019; Fischer et al. 2022); thus, exploring their mechanism in relationship with grain size regulation is important (Liang et al. 2021).

To identify grain width and yield quantitative trait locus (QTL), narrow-grain HX354 (*gw5*) and wide-grain ZH11 (*gw5*) accessions were selected to construct an F_2_ population (Fig. 1A). Employing the principle of RapMap (Zhang et al. 2021), a region on chromosome 10, implies the cosegregation standard of single-locus genetic models (Supplementary Figs. S1 and S2). By developing molecular markers and progeny testing of screened recombinants from an F_2_ population, *GWY10* was fine-mapped to 18.17 kb region (Fig. 1B; Supplementary Fig. S3). The region harbored 3 predicted ORFs, and only *ORF2* was differentially expressed. The haplotype Hap^HX354^ carries high expression and narrow grains, and Hap^ZH11^ exhibits low expression and wide grains (Supplementary Fig. S4; Supplementary Table S1). Thus, *ORF2* (*LOC_Os10g36650*) is a solid candidate gene underlying *Grain Weight and Yield on Chromosome 10* (*GWY10*) QTL. Comparative sequencing between ZH11 and HX354 identified 54 natural variations in *GWY10* (Fig. 1C), and *GWY10* exhibited a higher transcript level in young panicles of homozygous BB/HX354 (Supplementary Fig. S5), implying that grain width variation between both parents was due to differential expression levels of *GWY10*, likely caused by natural variations in their promoter.

**Figure 1.**
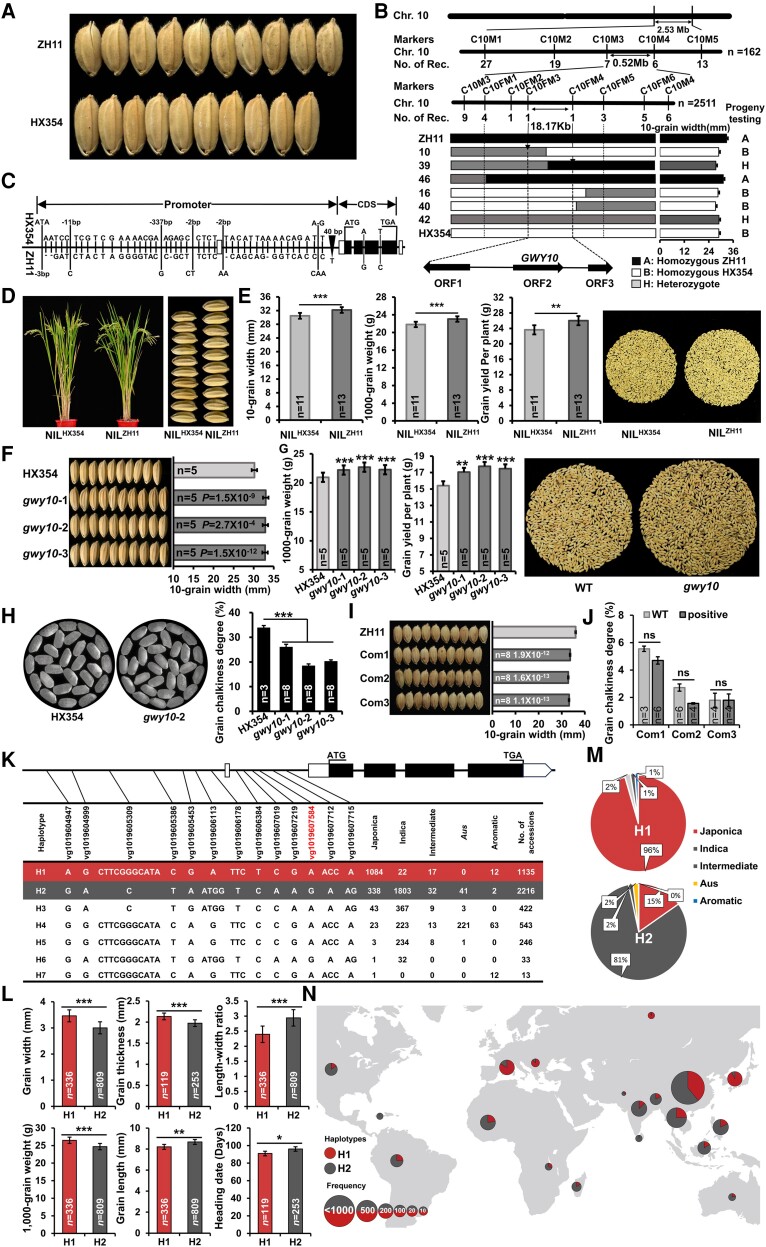
Identification of *GWY10* by map-based cloning for grain width and yield in rice. **A)** Grain width of ZH11 and HX354. **B)** Fine-mapping of *GWY10*. The name above the vertical line indicates molecular markers and the down number of recombinants between them. **C)** The structure and allelic variations of *GWY10*. **D)** Plant architecture and grain width of NIL^HX354^ and NIL^ZH11^. **E)** Grain width, weight, and yield per plant between NIL^HX354^ and NIL^ZH11^. Data are given as means ± Sd (*n* = 10). **F)** Grain width of *GWY10* knockout lines. **G)** Grain weight and yield between HX354 and knockout lines. **H)** Grain chalkiness between HX354 and *gwy10* lines. **I)** Grain width of T_1_ complementary lines. **J)** Grain chalkiness degree of complementary lines in T_1_ progenies. **K)** Seven haplotypes using 13 representative variations of *GWY10* in 4,726 rice accessions. **L)** Agronomic trait phenotype of H1 and H2 in 4,726 rice accessions. **M)***Indica–japonica* differentiation of major haplotypes H1 and H2. **N)** Geographical distribution of H1 and H2 haplotypes in 4,726 rice accessions. *Significant difference (*P* < 0.05). **Significant difference (*P* < 0.01). ***Significant difference (*P* < 0.001). ns, not significant difference. All data are given as means ± Sd. Two-tailed Student's *t* test was used to generate the *P* values. *n* represents the number of biologically independent experiments or accessions.

By investigating agronomic traits of *GWY10* near-isogenic line (NIL), we found a significant grain width difference between NIL^HX354^ and NIL^ZH11^. NIL^ZH11^ showed higher 1000-grain weight and grain yield than NIL^HX354^ by 5.59% and 10.03%, respectively, while other agronomic traits remained unchanged (Fig. 1, D and E; Supplementary Fig. S6). Moreover, NIL^HX354^ showed high transcript level in NIL^HX354^ in young panicles (Supplementary Fig. S7), signifying *GWY10* as a negative regulator of grain width.

Knockout mutants of *GWY10* in high expressing HX354 background showed 9.04% increase in grain width and 6.88% increase in 1000-grain weight, enhancing grain yield by 13.03% compared with wild type, without changes in other yield traits (Fig. 1, F and G; Supplementary Figs. S8 and S9). Grain width indirectly affects grain chalkiness and quality (Ren et al. 2023), while transparency of milled grains was greatly reduced in *gwy10*, showing a similar grain chalkiness rate to HX354 (Fig. 1H; Supplementary Fig. S10A). We generated complementary lines by introducing the stronger HX354 allele into ZH11 background (Supplementary Fig. S11), which significantly reduced grain width compared with wild-type plants without change in grain chalkiness (Fig. 1, I and J), implying the negative role of *GWY10* for grain width.

Haplotype analysis of natural variation of *GWY10* promoter by utilizing 4,726 accessions of rice worldwide (Zhao et al. 2021) identified 7 haplotypes (H1 to H7) (Fig. 1K; Supplementary Table S2). Two major haplotypes H1 and H2 marked significant differences in grain width, 1000-grain weight, grain length–width ratio, and grain length (Fig. 1L; Supplementary Fig. S12A). Subpopulations and geographical distribution analysis showed that 91% of accessions in H1 belong to *japonica* and majorly distributed in South Korea, Japan, and Europe, while 81% of accessions in H2 belong to *indica* in Asian and American regions (Fig. 1, M and N; Supplementary Fig. S12B), suggesting that different haplotypes of *GWY10* had significant *indica–japonica* differentiation and geographical distribution for different environmental adaptation, suggesting that *indica* and *japonica* had undergone different domestication processes.

To explore the relationship between *GWY10* and other grain width genes, we performed a coexpression profiling and detected *GWY10* expression in genetic materials of other grain width genes (Supplementary Figs. S13 and S14). We identified *cis*-regulatory elements/binding sites for transcription factors underlying natural variations in 2 kb *GWY10* promoter. We found that an single nucleotide polymorphism (SNP) (A^ZH11^ to G^HX354^) at −1,222 upstream of start codon (ATG) led to the formation of a G-box (CACGTG) in HX354 promoter (Fig. 2B), a sequence bound by *bZIP* transcription factor family. Correlation expression analysis of bZIPs between binding and nonbinding accessions of *GWY10* found OsbZIP47 (*LOC_Os06g15480*) as a solid candidate exhibiting high coexpression with *GWY10* (Fig. 2A; Supplementary Table S3) and involved in grain size regulation in rice (Hao et al. 2021). Electrophoretic mobility shift assays (EMSA) demonstrated that OsbZIP47 has a high binding affinity with *GWY10*^HX354^ (binding) allele promoter than *GWY10*^ZH11^ (nonbinding) (Fig. 2, B and C). Furthermore, chromatin immunoprecipitation-quantitative PCR (ChIP-qPCR) assays showed high fold enrichment for promoter containing the G-box fragment in binding allele compared with nonbinding allele (Fig. 2D), demonstrating that OsbZIP47 probably causes *GWY10* differential expression. Moreover, compared with the nonbinding promoter activity, the LUC/REN activity of the binding promoter was increased in the presence of OsbZIP47 (Fig. 2E). *GWY10* exhibited higher expression in binding accessions than nonbinding accessions of rice mini-core collection (Fig. 2F). Furthermore, *GWY10* showed a significant positive correlation between high and low expression accessions of *OzbZIP47* (Fig. 2, F and G). We also found that *GWY10* expression was significantly upregulated in overexpressing *OsbZIP47* plants (Fig. 2H). These results indicated that *OsbZIP47* acts as a transcription factor to regulate *GWY10* differential expression.

**Figure 2.**
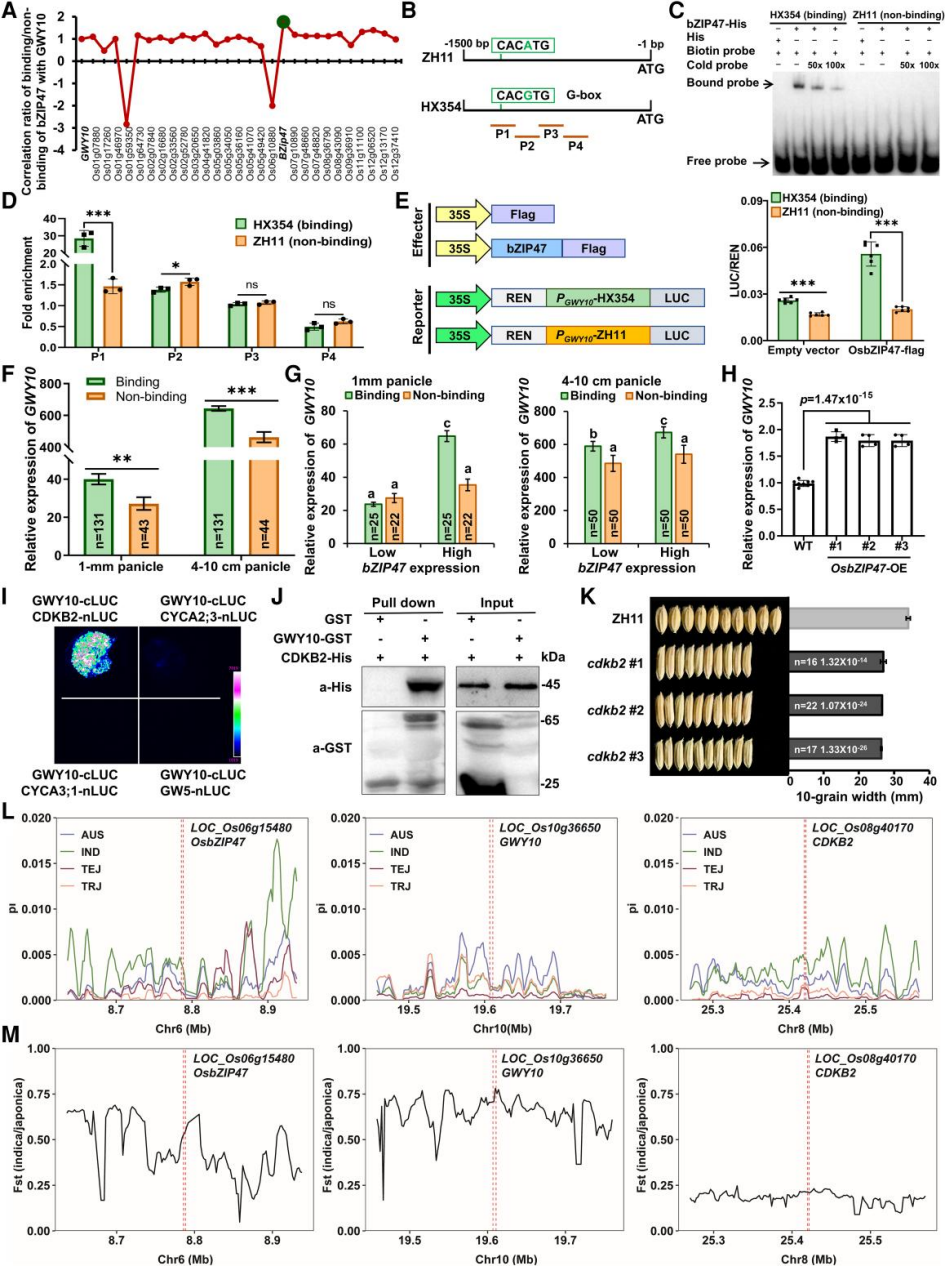
Regulation of *GWY10* by OsbZIP47, interaction of GWY10 by CDKB2, and nucleotide diversity of *OsbZIP47*, *GWY10*, and *CDKB2*. **A)** Coexpression analysis of OsbZIP transcription factors with *GWY10*. **B)** Probes in promoters of GWY10 for OsbZIP47-mediated EMSA (green box) and ChIP-qPCR (orange line). **C)** Electrophoretic mobility shift and **D)** ChIP-qPCR assays for the differential binding affinity of OsbZIP47 with *GWY10* promoters in ZH11 and HX354, respectively. P1 to P4 represents different fragments of *GWY10* promoter. Data presented are the means ± Sd (*n* = 3). **E)** LUC/REN activity of *GWY10* binding and nonbinding promoters bound by OsbZIP47. Data presented are the means ± Sd (*n* = 6). **F)***GWY10* expression between binding and nonbinding accessions of rice mini-core collection. **G)***GWY10* expression in binding and nonbinding accessions with low or high expression of *OsbZIP47* in rice mini-core collection. **H)***GWY10* expression levels in *OsbZIP47* overexpression lines. Data presented are the means ± Sd (*n* = 4). **I)** Split-LUC assays for screening the interaction of GWY10 with cyclin-related proteins (CDKB2, CYCA2; 3, CYCA3; 1) and GW5. Magenta was used instead of red to avoid red/green combinations for people with colorblindness. **J)** Confirmation of GWY10 interaction with CDKB2 by pull-down assays. **K)** Grain width of 3 independent CRISPR lines of *cdkb2* in ZH11. **L)** Nucleotide diversity of *OsbZIP47*, *GWY10*, and *CDKB2* and their surrounding 100 kb region in different rice subgroups of the rice mini-core collection. AUS, IND, TEJ, and TRJ represent *aus*, *Indica*, *Temperate japonica*, and *Tropical japonica* accessions, respectively. **M)** The Tajima's *D* values (Fst) of *OsbZIP47*, *GWY10*, and *CDKB2* genomic sequences in diverse accessions of the rice mini-core collection. The red-dotted line shows the location of *OsbZIP47*, *GWY10*, and *CDKB2*. *Significant difference (*P* < 0.05). **Significant difference (*P* < 0.01). ***Significant difference (*P* < 0.001). ns, not significant difference. All data are given as means ± Sd. Two-tailed Student's *t* test was used to generate the *P* values. *n* represents the number of biologically independent experiments or accessions.

*GWY10* encodes a conserved actin protein, participates biological processes of eukaryotes (Dogterom and Koenderink 2019; Gu and Rasmussen 2022), and acts as an important factor during plant cell division (Caillaud 2022). To further explore the *GWY10* molecular mechanism underlying grain size regulation, we screened GWY10-interacting proteins from cyclins (CYCAs) and CDKs, which regulate eukaryotic cell cycle and cell division (Dogterom and Koenderink 2019). The results confirmed physical interaction between GWY10 and CDKB2 (Fig. 1, I and J; Supplementary Fig. S15). Grain width of the knockout mutant *cdkb2* in ZH11 carrying *GWY10* weak allele with wide grains was significantly narrowed (Fig. 2K; Supplementary Fig. S16). The results support that *GWY10* and *CDKB2* act in a common pathway to control grain width and yield in rice.

Given the crucial roles of *OsbZIP47*, *GWY10*, and *CDKB2* in regulating grain width and yield of rice, we studied their variations in rice germplasm (Wang et al. 2016). Comparison of *OsbZIP47* and *GWY10* sequences in different accessions showed low nucleotide diversity (*P* < 0.005) (Fig. 2L) and selective scanning at 2 loci, and negative Fst values were observed mainly in *indica* but not *japonica* subgroups (Fig. 2M), implying that *OsbZIP47* and *GWY10* underwent differential selection during *indica* rice adaptation, while *CDKB2* did not experience such significant selection.

Grain size is the key agronomic trait for crop yield and breeding. Our findings suggest a previously unknown working model for OsbZIP47–GWY10–CDKB2-mediated control of grain size and yield in rice. The OsbZIP47 acted as a transcription factor and regulated its expression by differentially binding to *GWY10* promoters, while conserved actin GWY10 may function in a common pathway with a conserved CKDB2 to control grain size and yield without quality penalty in rice. The natural variations of *GWY10* from the *japonica* haplotype will help in improving the grain size and yield in future breeding efforts for rice and other crops.

## Supplementary Material

kiae456_Supplementary_Data

## Data Availability

The data and materials for this study presented in the manuscript and supplementary files are available from corresponding author on request.

## Supplementary data

The following materials are available in the online version of this article.

**Supplementary Figure S1.** Preliminary mapping of *GWY10*.

**Supplementary Figure S2.** Grain width segregation between AA/ZH11 and BB/HX354 homozygous genotypes of the F_2_ population.

**Supplementary Figure S3.** Genotyping by PARMS technology for recombinant screening.

**Supplementary Figure S4.** Grain width and expression of Hap^HX354^ and Hap^ZH11^ of 3 ORFs in the mini-core collection of 533 accessions.

**Supplementary Figure S5.** Expression of *GWY10* between AA/ZH11 and BB/HX354 homozygous lines of the F_2_ population.

**Supplementary Figure S6.** Comparison of grain length, number of effective tillers and number of filled grains between NIL^HX354^ and NIL^ZH11^ of *GWY10*.

**Supplementary Figure S7.** *GWY10* transcript levels in different tissues between 2 NILs.

**Supplementary Figure S8.** Phenotypes and genotypes of CRISPR lines of *GWY10*.

**Supplementary Figure S9.** Grain length, number of panicle, and number of filled grains of between HX354 and the T_1_ progenies for *gwy10* knockout lines.

**Supplementary Figure S10.** Grain chalkiness rate of *gwy10* knockout lines and the T_1_ progenies for *GWY10* complementary.

**Supplementary Figure S11.** Grain width of complementation lines for *GWY10*.

**Supplementary Figure S12.** Various agronomic traits between 2 haplotypes H1 and H1 of *GWY10* in 533 accessions and evolution of *GWY10* about grain width in rice.

**Supplementary Figure S13.** The correlation coefficients of expression levels among *GWY10* and grain width genes.

**Supplementary Figure S14.** *GWY10* transcript levels in various genetic materials of grain width genes.

**Supplementary Figure S15.** Split luciferase complementation assay for screening various interacting proteins with GWY10.

**Supplementary Figure S16.** Grain width and genotypes of CRISPR lines of *CDKB2*.

**Supplementary materials and methods.**

**Supplementary Table S1.** Mini-core collection of 533 rice accessions data representing natural variation, 1 and 4 cm panicle expression of ORF1, ORF2, and ORF3.

**Supplementary Table S2.** Agronomic traits, geographical distribution, and natural variations *GWY10* from the rice mini-core collection with 4,726 diverse accessions across the world.

**Supplementary Table S3.** Expressions of bZIP transcription factors family members in 1 mm panicle from the rice mini-core collection with 533 accessions for correlation analysis with GWY10.

**Supplementary Table S4.** Primer pairs used for QTL mapping, sequencing, expression vector construction, and functional analysis.

## Dive Curated Terms

The following phenotypic, genotypic, and functional terms are of significance to the work described in this paper:

grain width Planteome: TO:0000402

grain size Planteome: TO:0000397
