# Supplementary material for: The quantitative trait locus GWY10 controls rice grain width and yield
Source: Plant Physiol. 2024 Aug 29;196(4):2286–90. doi: 10.1093/plphys/kiae456 (PMC11637765; doi:10.1093/plphys/kiae456)
Supplement: kiae456_Supplementary_Data [file kiae456_supplementary_data.zip › suppdatafinal.docx]

**
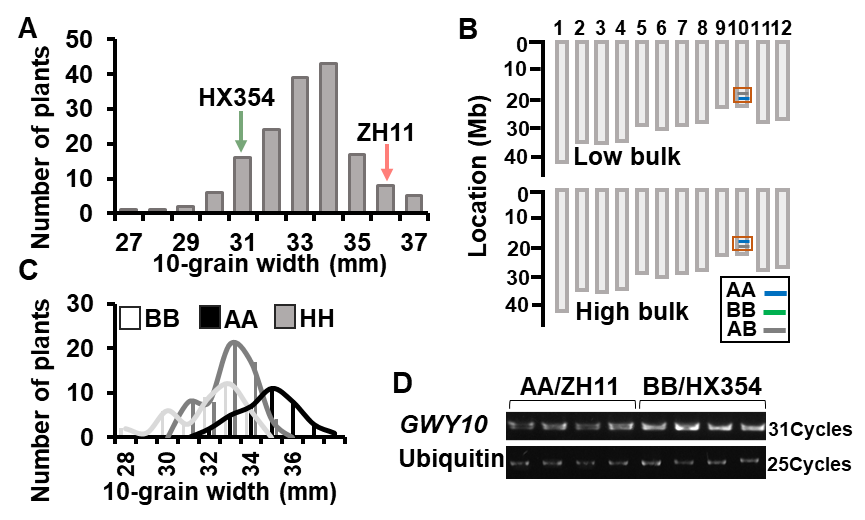
**

**Supplementary Figure S1. Preliminary mapping of *GWY10*.** (A) Grain width distribution of the F2 population. (B) Identification of GWY10 by RICE6K SNP Array coupled with BSA of two extreme bulks. GWY10 is marked by the orange rectangle on chromosome 10. (C) QTL validation by co-segregation analysis of RapMap. AA and BB represent the homozygous genotypes with high-value, low-value grain width, and HH the heterozygous genotype, respectively. (D) RT-PCR of GWY10 in young panicles between homozygous AA/ZH11 and BB/HX354 lines of the F2 population.

**
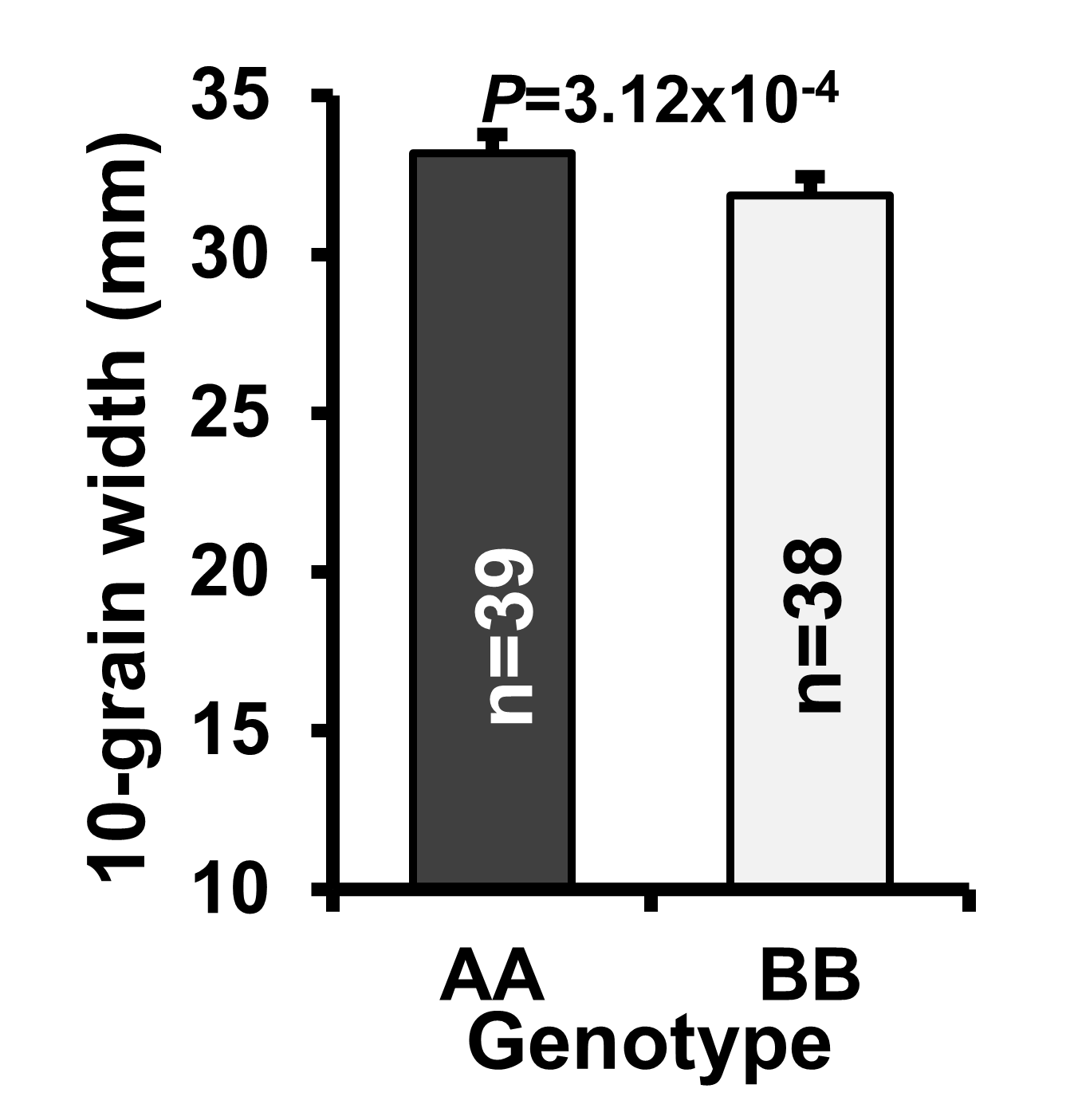
**

**Supplementary Figure S2. Grain-width segregation between AA/ZH11 and BB/HX354 homozygous genotypes of the F_2_ population.** n is the number of individual plants. All the *P* values were produced by the two-tailed *t*-tests. Data are shown as means ± SD.


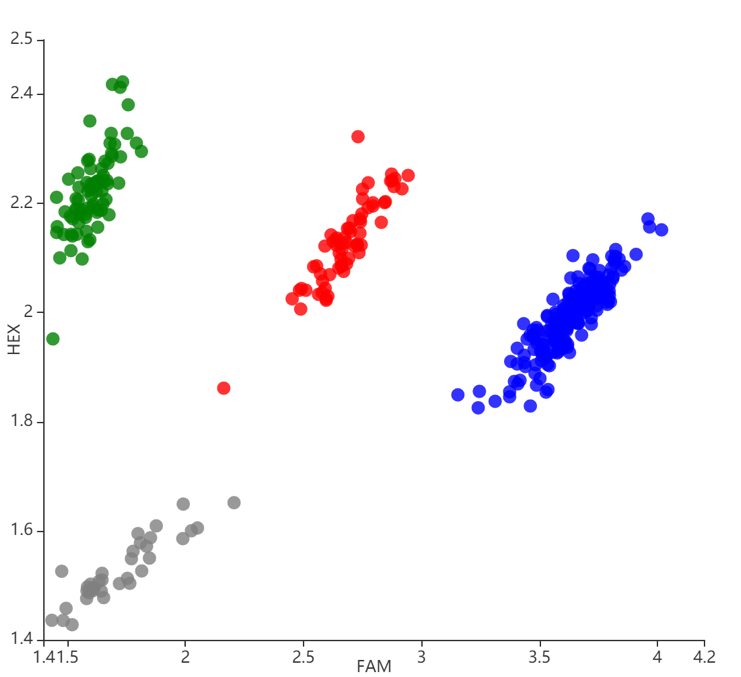


**Supplementary Figure S3. Genotyping by PARMS technology for recombinant screening.** The green (extreme upper left corner) and blue (extreme right) dots represent the homozygous genotype (AA and BB) while the red (middle) dots represent the heterozygous genotype (AB). The grey (extreme lower left corner) dots correspond to NTC (non-template control).

**
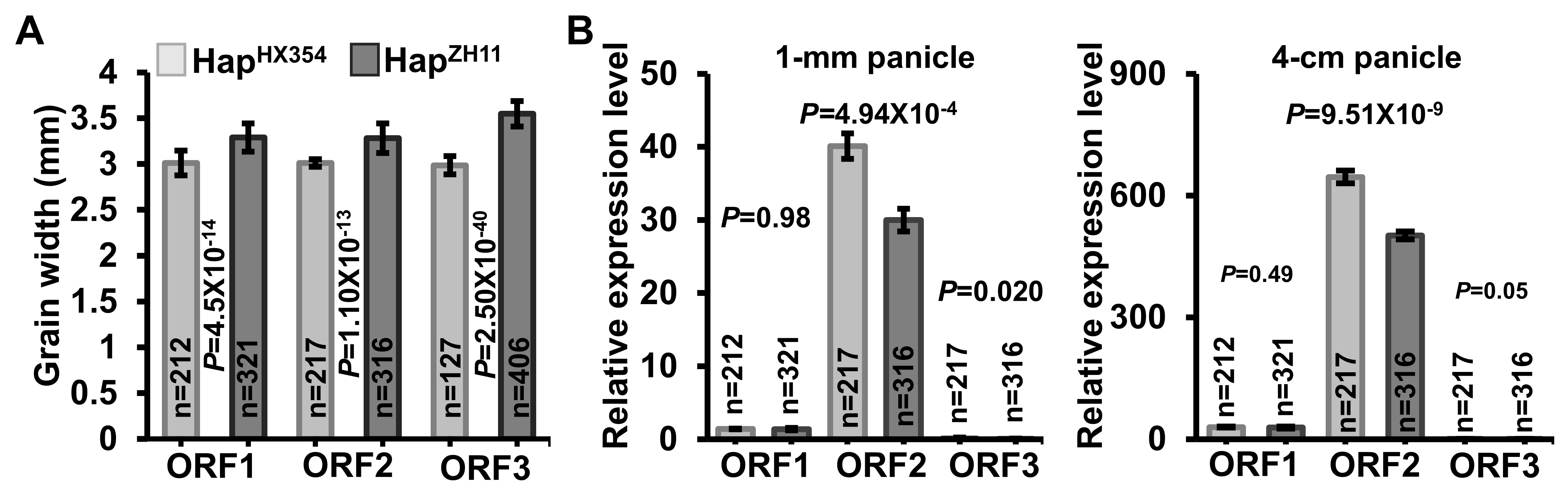
**

**Supplementary Figure S4. Grain width and expression of Hap^HX354^ and Hap^ZH11^ of three ORFs in the mini-core collection of 533 accessions.** (**A**) Phenotype effects of two different haplotypes Hap^ZH11^ and Hap^HX354^ of three ORF in the mini-core collection of 533 accessions. (**B**) Comparative differential expression analysis in 1-mm panicles between Hap^ZH11^ and Hap^HX354^ in the mini-core collection. n is the number of individual plants. All the *P* values were produced by the two-tailed *t*-tests. Data are shown as means ± SD.


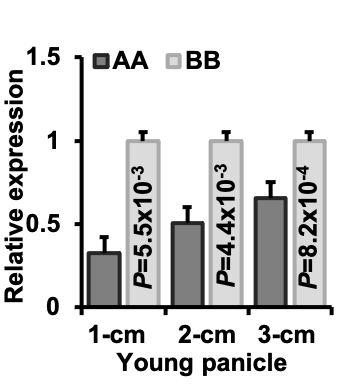


**Supplementary Figure S5. Expression of GWY10 between AA/ZH11 and BB/HX354 homozygous lines of the F_2_ population.** All the *P* values were produced by the two-tailed *t*-tests. Data are shown as means ± SD (n=3).

**
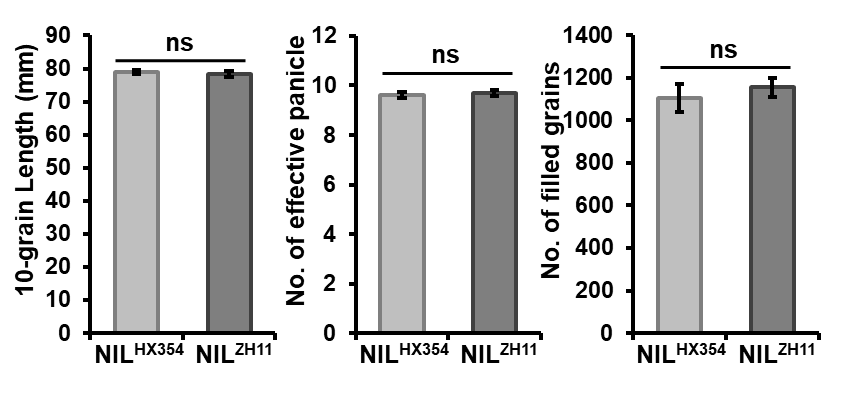
**

**Supplementary Figure S6. Comparison of grain length, number of effective tillers and number of filled grains between NIL^HX354^ and NIL^ZH11^ of *GWY10*.** ns, not significant difference (*t*-test). Data are shown as means ± SD (n=10). All the *P* values were produced by the two-tailed *t*-tests.

**
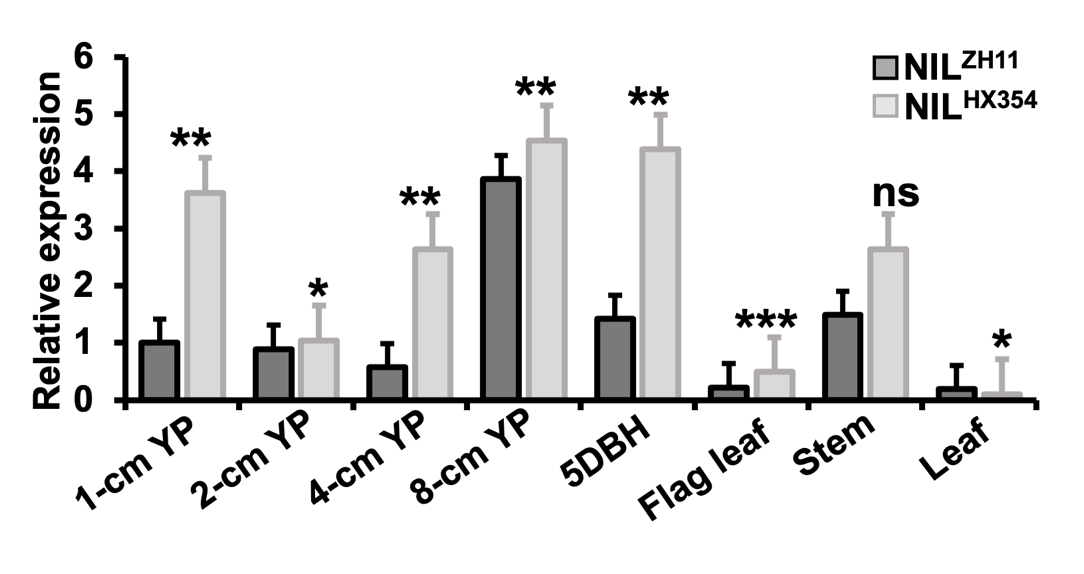
**

**Supplementary Figure S7. *GWY10* transcript levels in different tissues between two NILs.** YP, young panicles with the average lengths; 5DBH, hulls at 5 days before heading. All abundances shown are relative to those of NIL^ZH11^. *Significant difference (*P* < 0.05). **significant difference (*P* < 0.01, *t*-test). ***significant difference (*P* < 0.001, *t*-test). ns: not significant difference (*t*-test). Rice ubiquitin gene was used as a control. Data are shown as means ± SD (n=3).. All the *P* values were produced by the two-tailed *t*-tests.


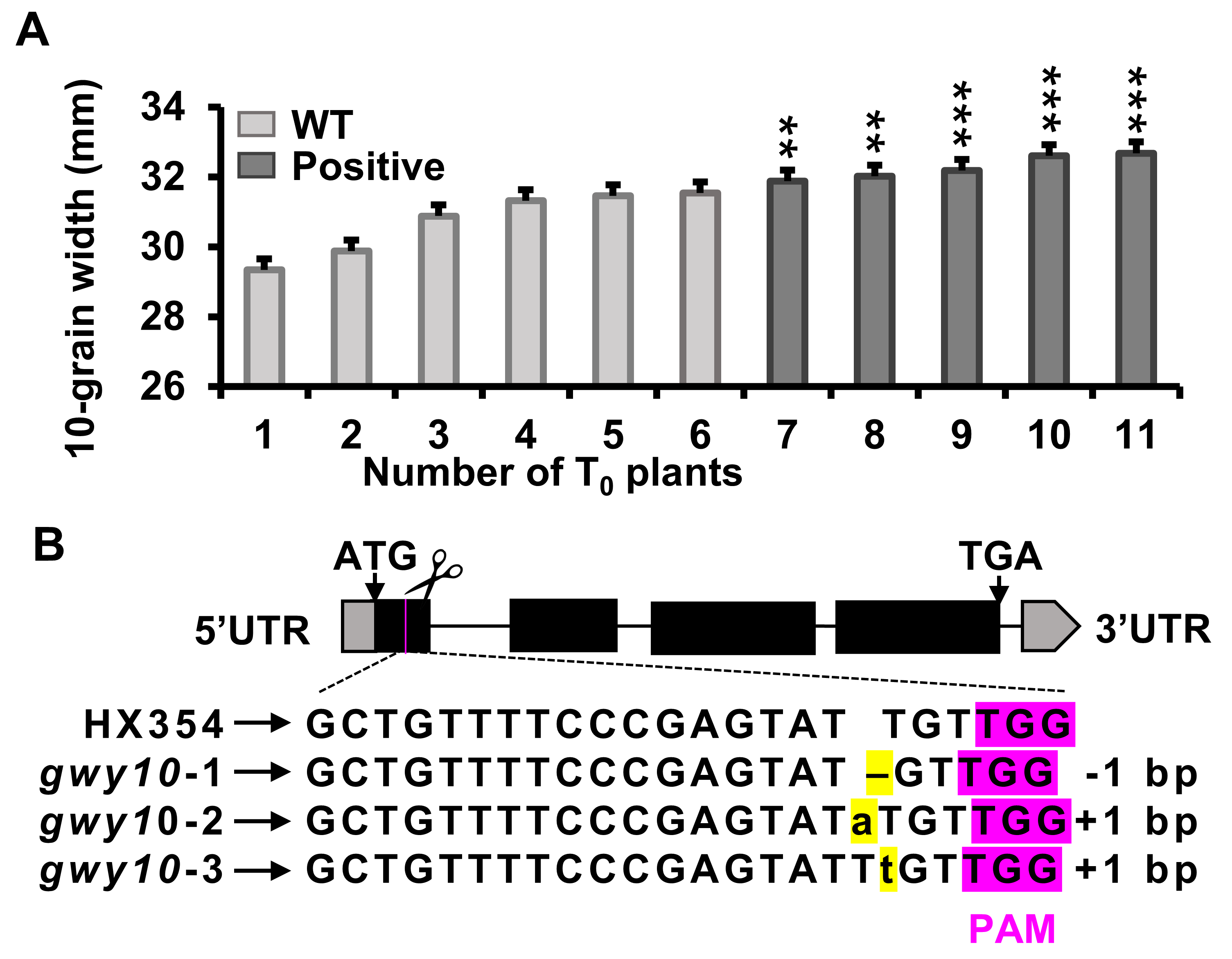


**Supplementary Figure S8. Phenotypes and genotypes of CRISPR lines of *GWY10*.** (**A**) Distribution of grain width in the independent individuals of edited positive and unedited WT for *GWY10*. All data are shown as mean value ± SD. **significant difference (*P* < 0.01, *t*-test). ***significant difference (*P* < 0.001, *t*-test). All the *P* values were produced by the two-tailed *t*-tests. (**B**) Frameshift mutation of three independent *gwy10* knockout lines. The magenta box represents the PAM sequence.


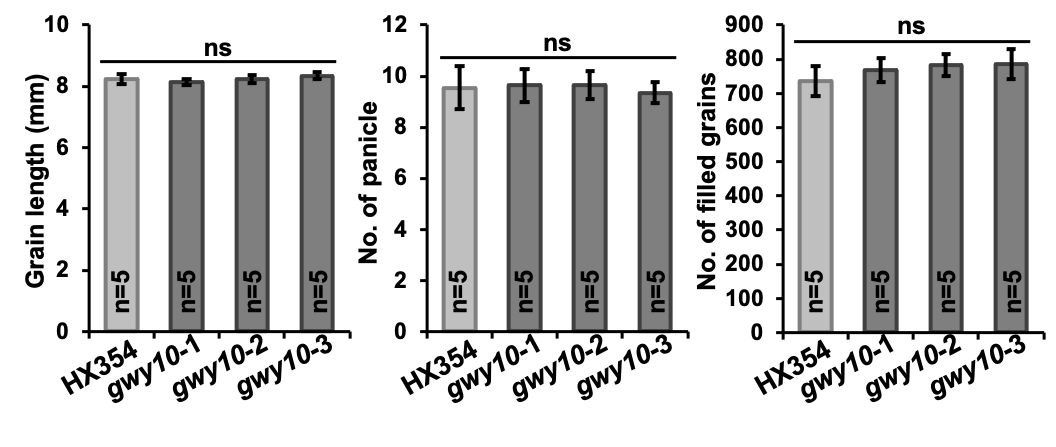
 **Supplementary Figure S9. Grain length, number of panicle and number of filled grains of between HX354 and the T_1_ progenies for *gwy10* knockout lines**. ns: not significant difference (*t*-test). *P* value is obtained by two-tailed *t*-test; n is the number of individual plants. Data shown as Means ± SD.


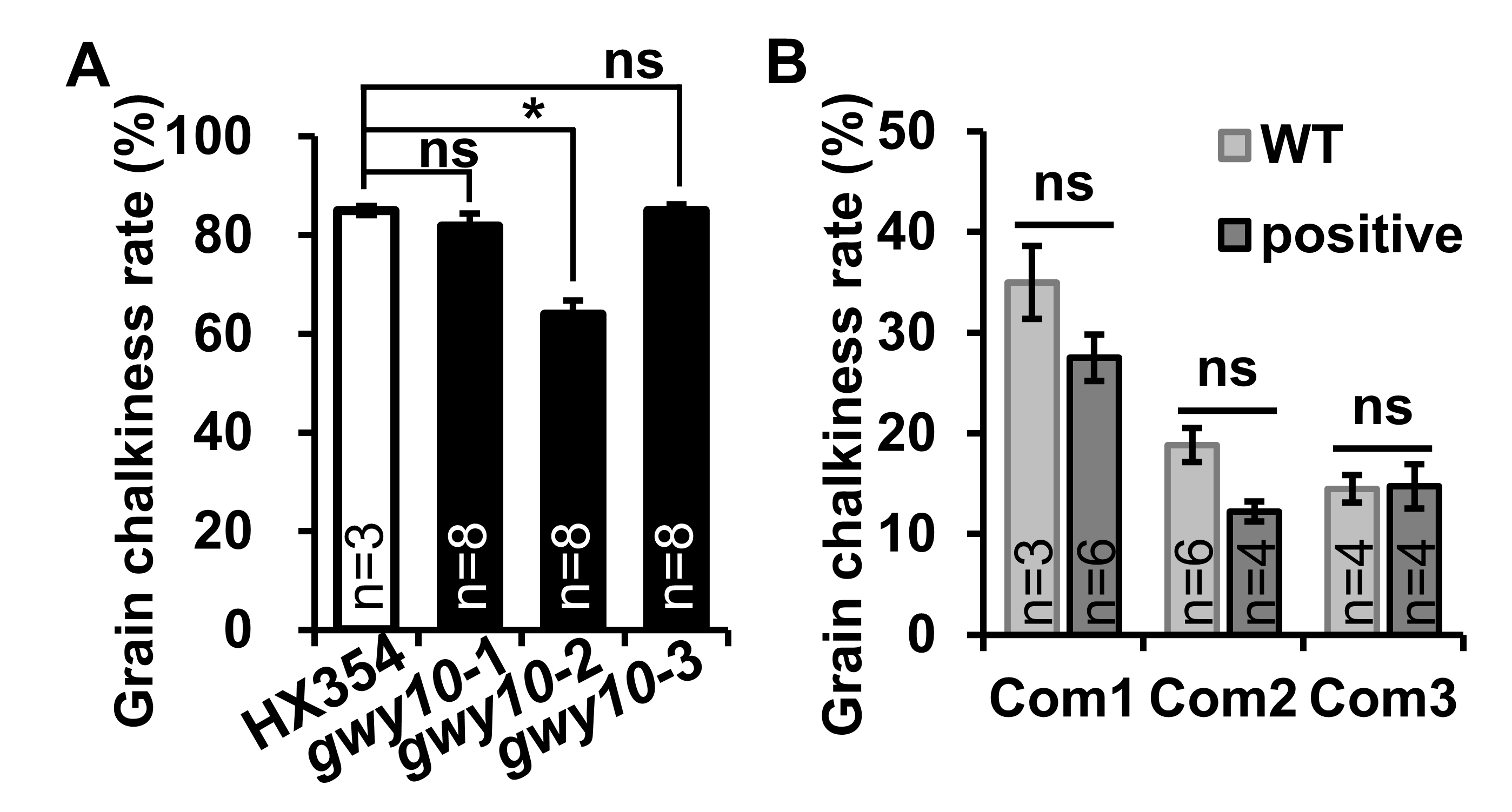


**Supplementary Figure S10. Grain chalkiness rate of *gwy10* knockout lines and the T_1_ progenies for *GWY10* complementary.** (A) Grain chalkiness rate between HX354 and *gwy10* knockout lines. (B) Statistics of grain chalkiness rate of T_1_ progenies for *GWY10* complementary. *Significant difference (*P* < 0.05). ns: not significant difference (*t*-test). *P* value is obtained by two-tailed *t*-test; n is the number of individual plants. Data shown as Means ± SD.

**
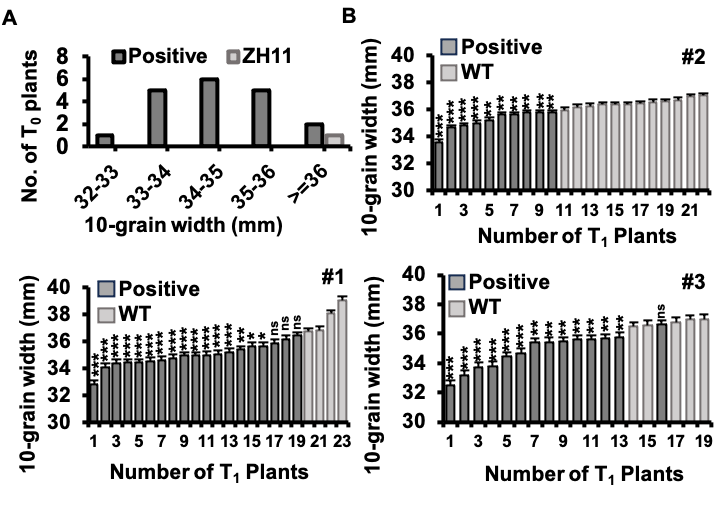
 Supplementary Figure S11. Grain width of complementation lines for *GWY10*.** **(A)** Phenotype distribution of all the independent complementation lines for *GWY10* in T_0_. **(B)** Co-segregation tests of genotype and grain-width phenotype in the three independent complementary lines of *GWY10* in T_1_. *Significant difference (*P* < 0.05). **significant difference (*P* < 0.01, *t*-test). ***significant difference (*P* < 0.001, *t*-test). ns: not significant difference (*t*-test). Values are shown as means ± SD (*n* = 3).


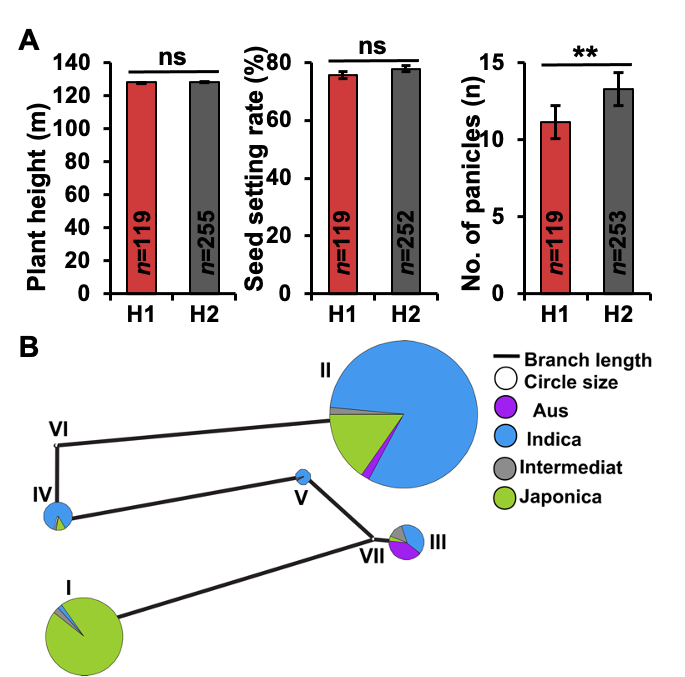


**Supplementary Figure S12. Various agronomic traits between two haplotypes H1 and H1 of GWY10 in 533 accessions and evolution of *GWY10* about grain width in rice. (A)** Comparison of plant height, seed setting rate, and number of panicles between H1 and H2 in the mini-core of 533 accessions, respectively. **(B)** Haplotype network analysis of *GWY10* using the representative variations of its promoter region. *Indica* group represents *Indica*-I, *Indica*-II, *Indica*-III, and *Indica* Intermediate. *Japonica* group includes *Temperate-japonica*, *Tropical-japonica* and *Japonica* Intermediate. The intermediate group includes *Aromatic* and other accessions. Each pie chart represents one unique haplotype, the haplotype groups are labeled with Roman numerals. **significant difference (*P* < 0.01, *t*-test). ns: not significant difference (*t*-test). Data are shown as means ± SD. *P* value is obtained by two-tailed *t*-test.


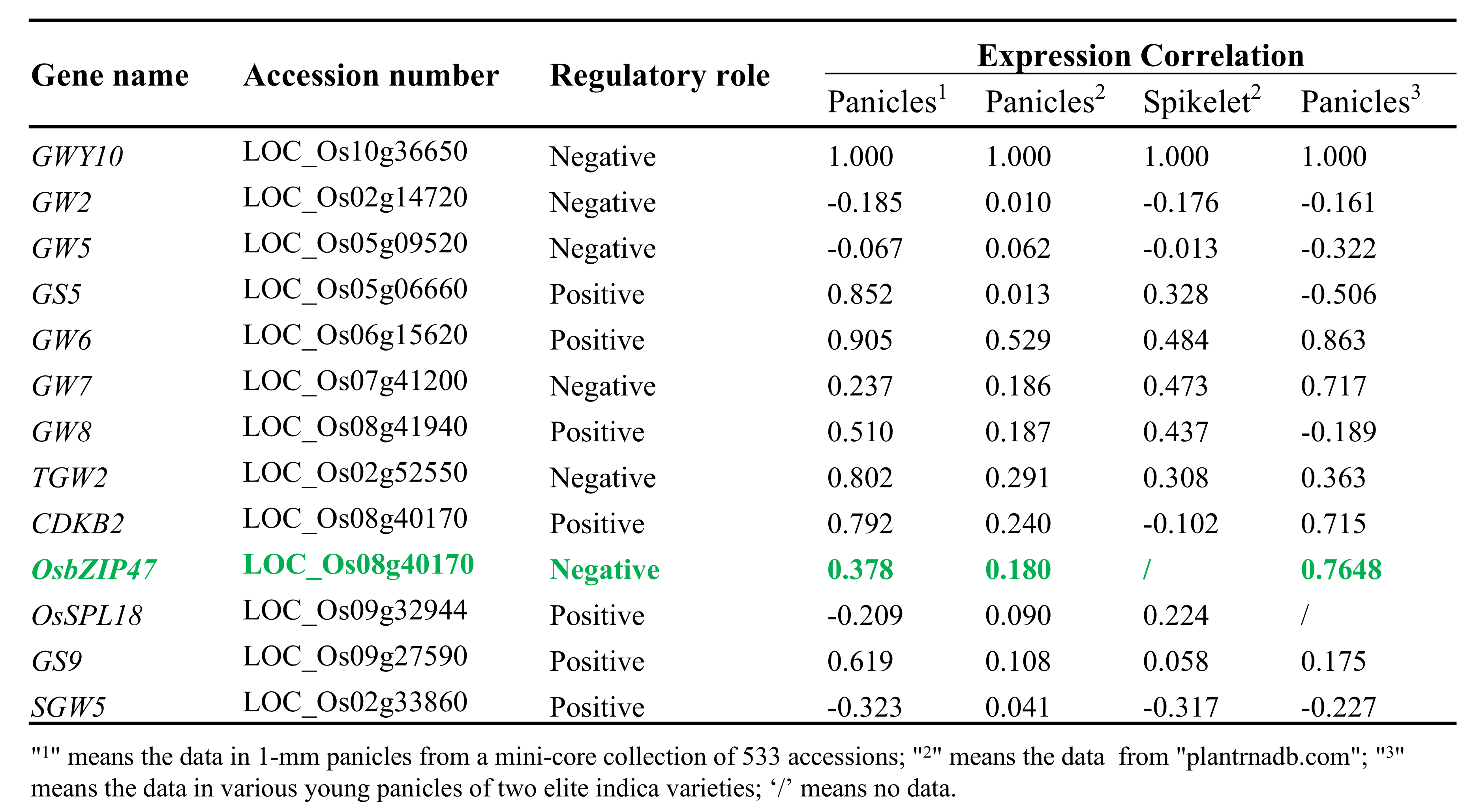


**Supplementary Figure S13. The correlation coefficients of expression levels among *GWY10* and grain width genes.**

**
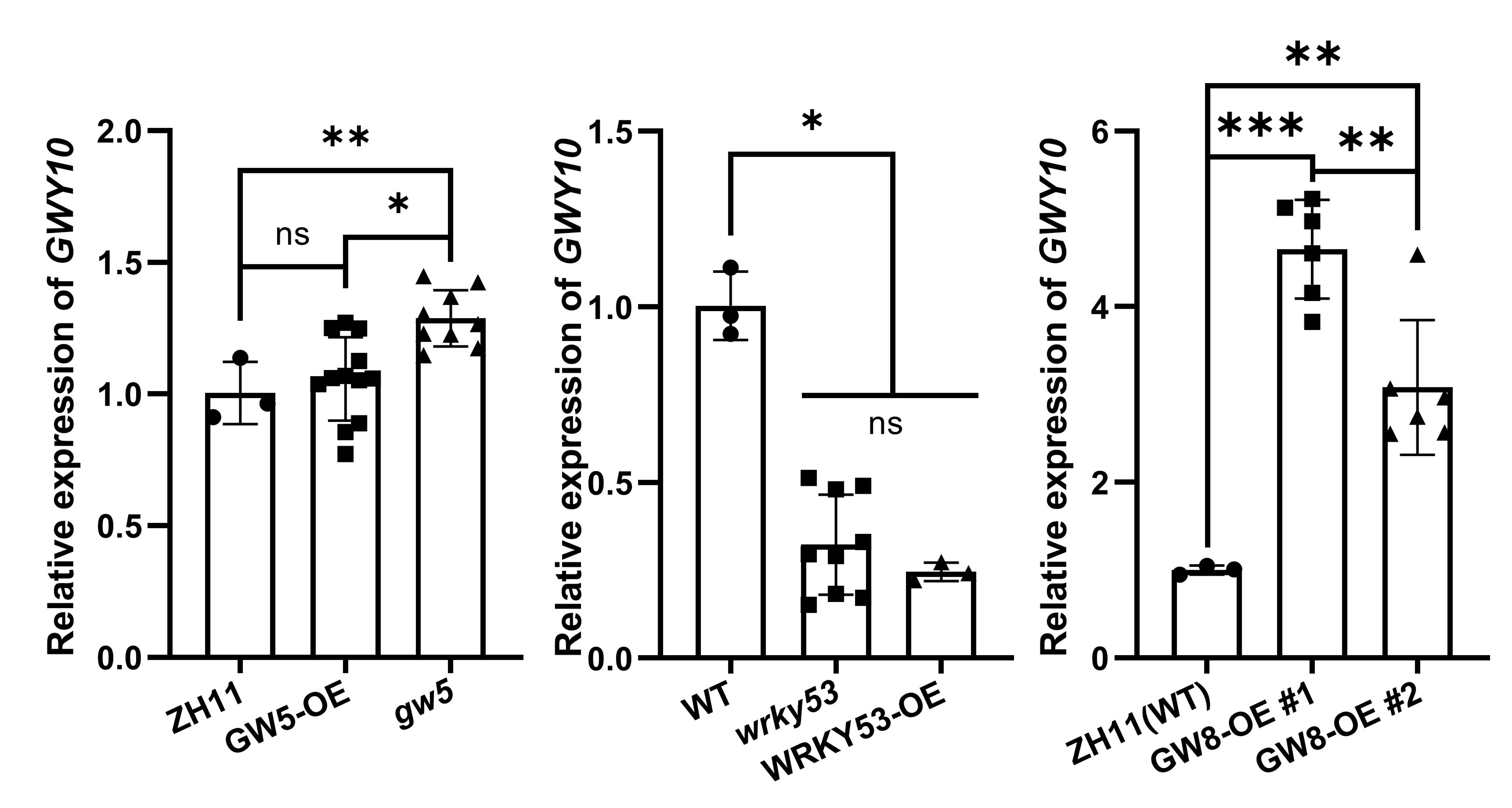
**

**Supplementary Figure S14. *GWY10* transcript levels in various genetic materials of grain width genes.** *Significant difference (*P* < 0.05). **significant difference (*P* < 0.01, *t*-test). ***significant difference (*P* < 0.001, *t*-test). ns: not significant difference. Data are shown as means ± SD. The two-tailed student’s *t*-test was used to generate the *P* values.

**
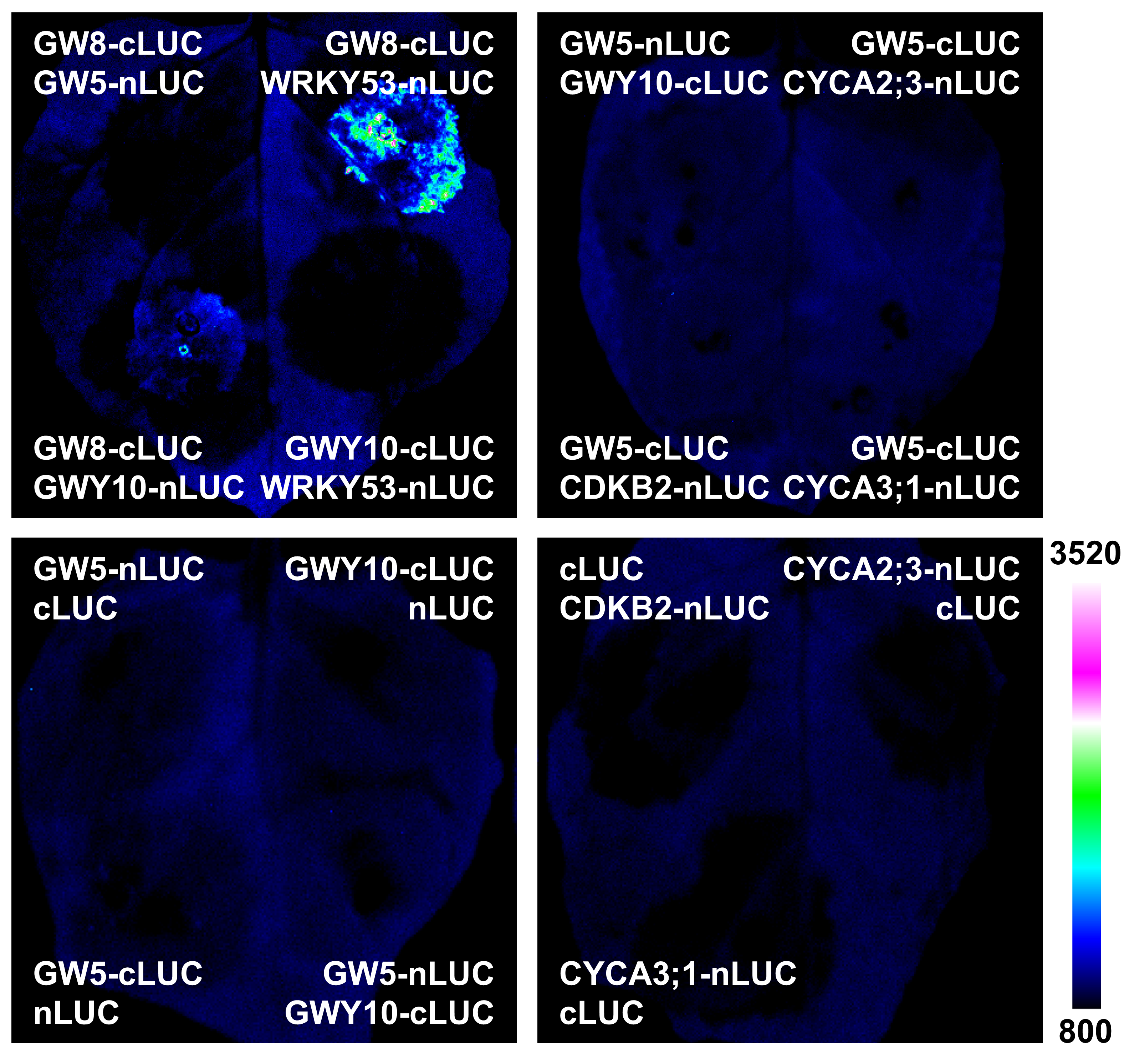
**

**Supplementary Figure S15. Split luciferase complementation assay for screening various interacting proteins with GWY10.** All experiments were repeated independently twice with similar results. Magenta was used instead of red to avoid red/green combinations for people with colorblindness.


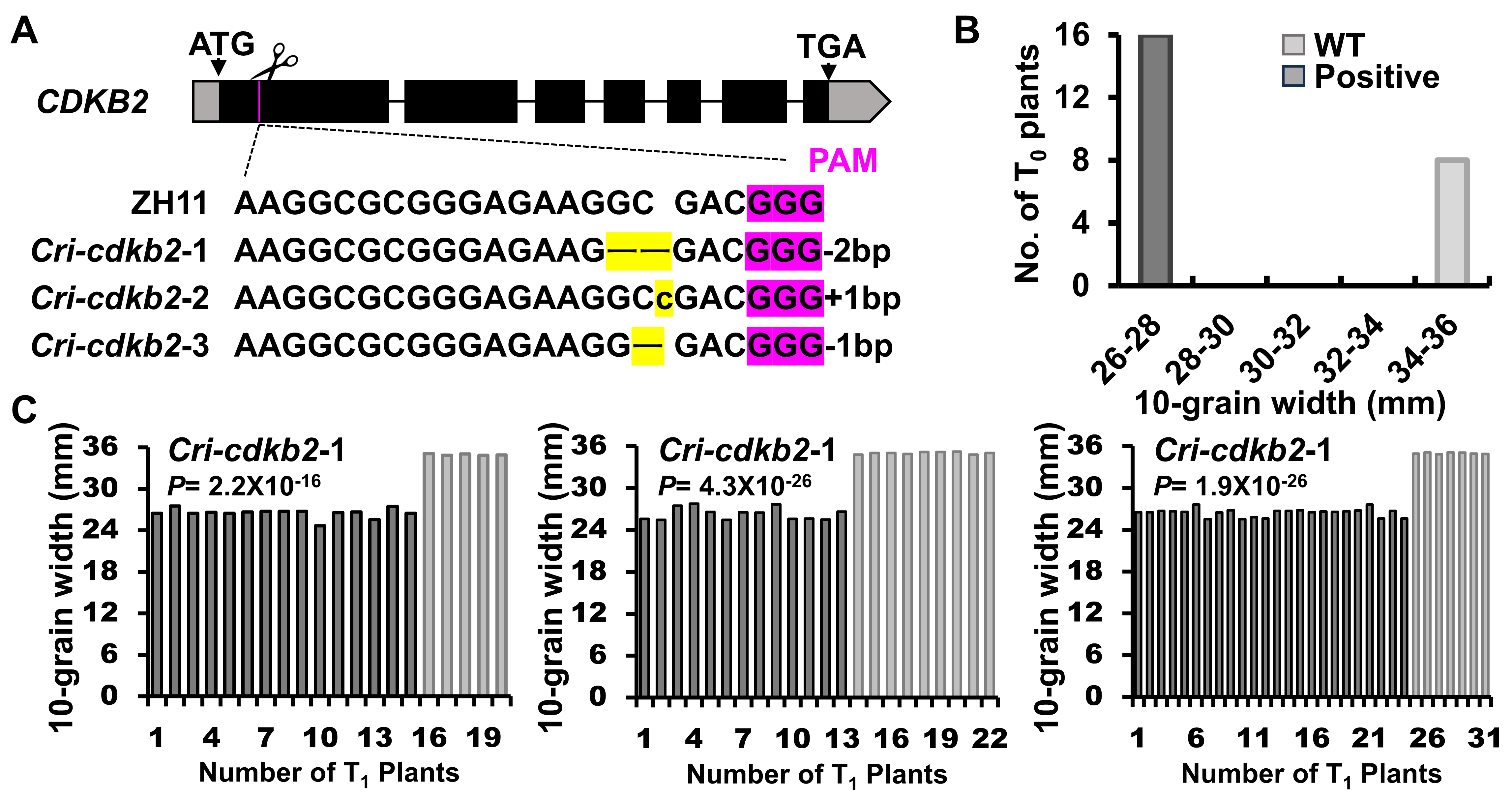


**Supplementary Figure S16. Grain width and genotypes of CRISPR lines of *CDKB2*.** (**A**) The sgRNA target site and genotypes of the three independent *cdkb2* knockout lines by CRISPR/Cas9 in ZH11 background. (**B**) Grain width distribution of *cdkb2* knockout lines in T_0_ progenies and (**C**) co-segregation analysis of grain width and genotype of three independent knockout lines of *cdkb2* in T_1_ progenies. All the *P* values were produced by the two-tailed *t*-tests. The magenta box represents the PAM sequence.

**Supplemental methods and materials**

**Genetic population construction.** The parental lines ZH11 (*Japonica*) and HX354 (an *Indic*a) rice varieties carrying the non-functional genotype of major grain width gene *GW5* were selected from 533 accessions mini-core collection to develop F_2_ population **(Supplementary Table S1).** ZH11 a wide grain variety as a female parent crossed with HX354 a narrow grain variety as a male. The true hybrid progeny was harvested and an F_2_ population of 162 individuals was constructed following the self-pollination.

**Plant material, growing conditions, and phenotype measurement.** All genetic and transgenic material was planted at Huazhong Agricultural University research stations in Wuhan and Lingshui cities. The 25-days old seedlings were transplanted in the paddy field, and the distance between the plants was maintained at 16.5 cm. Standard plantation practices were applied such as disease and insect-pests control, irrigation, and fertilizer application. Mature grains of genetic and transgenic material were harvested and air-dried. The grain-width, grain length was measured by an electronic vernier caliper. Total grain weight was calculated and converted to 1000-grain weight and grain yield per plant from two NIL lines was determined by three replications planted in random complete block designs The grain quality attributes were calculated by naked eye observation. Bulk segregant analysis. Following the grain width measurement of 162 individuals of the F_2_ population, two bulks with extreme grain width were constructed: a low bulk carrying 21 individuals comprising of narrow grain width of less than 31 mm (10-grain width) and a high bulk carrying 26 individuals comprising of wide grain width of more than 35 mm (10-grain width). Seeds of two bulks (each plant contributed 10 seeds) were separately germinated in a growth chamber with optimized conditions such as day and night temperatures maintained at 30 ºC and 25 ºC respectively and the relative humidity of the growth chamber was about 75%. The leaves of 10-14 days old seedlings were used for DNA extraction by the CTAB method. Two extreme bulks were detected by the BSA method combined with the RICE6K SNP Array by China National Seed Group Co., Ltd.

**RapMap and map-based cloning of *GWY10*.** After QTL region detection by bulk segregant analysis (BSA) based on the RICE6K SNP Array, we designed a series of molecular markers based on polymorphic (InDel) variations between ZH11 and HX354 retrieved from RiceVarMap database (http://ricevarmap2.ncpgr.cn/v1/). A total of 162 F_2_ plants derived from the cross of ZH11 and HX354 were used for genetic analysis and mapping of *GWY10* at chromosome 10. For further fine mapping additional 2511 recombinants were screened by PARMS (penta-primer amplification refractory mutation), a KASP (Kompetitive Allele Specific PCR) like genotyping method by Wuhan Gentides Biotech Co., Ltd., Wuhan. The screened recombinants were planted (each recombinant comprising 36 plants for progeny testing. The genotype was inferred as: the progenies carrying high grain-width value without segregation were homozygous AA alleles ZH11, while progenies exhibiting low-value grain width without any segregation were homozygous BB alleles from HX354, whereas, if the grain-width phenotype of the progeny individual is segregated, the two alleles of *GWY10* of the recombinant individual were considered as heterozygous HH. All molecular markers and primers applied in this study are listed in (**Supplementary Table S4)**.

**Construction of complementary and knockout transgenic lines.** To validate the function of *GWY10* on grain width, the coding sequence together with the 4.1-kb upstream region of the start codon as a promoter of *GWY10* was amplified from the HX354 parental DNA and inserted into the plant binary vector pCAMBIA 1301-Flag between the EcoRI and KpnI sites using Gibson assembly. The cloned vector was verified by sequencing and introduced into ZH11 by *Agrobacterium-mediated* transformation. The InDel marker between ZH11 and HX354 was used to detect the positive complementary lines on PAGE.

CRISPR/Cas9 technology was applied to generate *gwy10* and *cdkb2* knockout plants. The gene-specific guide sequence (sgRNA) for the construction of the loss of function alleles of *GWY10* and *CDKB2* was designed and integrated into the *OsU6* and *OsU3* promoters respectively by an overlapping PCR. Then, the sgRNA transcriptional system was inserted into the pCXUN-CAS9 plasmid at the KpnI site through Gibson assembly. The vectors for *GWY10* and *CDKB2* were introduced into HX354 and ZH11 respectively by *Agrobacterium*-mediated transformation. The mutation in transgenic plants was detected by polyacrylamide gel electrophoresis technology (PAGE) and verified by NGS sequencing by amplifying the target sequence with flanking primers. All primers for vector construction and sequencing are listed in **Supplementary Tabl**e **S4**

**NIL development for *GWY10*.** The nearly isogenic lines (NIL^HX354^ and NIL^ZH11^) for *GWY10* were generated by marker-assisted selection. The HX354 was used as a recurrent parent and was backcrossed with heterozygous (HH) plants similar to HX354. The backcross was repeated several times until the plant type of the true hybrid same as the recurrent parent was obtained. The genotypes of two segregated homozygous NIL^HX354^ and NIL^ZH11^ were identified and planted in the paddy field of the experimental research station of Huazhong Agricultural University in Wuhan. Fully matured grains were harvested for further agronomic trait analysis.

**Natural variations, haplotype analysis, and genetic diversity.** The natural variations between ZH11 and HX354 of *GWY10* 2-kb promoter, coding, and 0.5-kb downstream regions from 533/4726 rice accessions were retrieved from the RiceVarMap v2.0 database (<http://ricevarmap.ncpgr.cn>). The representative natural variations were utilized to perform the haplotype analysis and agronomic traits and expression analysis was performed between two major haplotypes. To perform haplotype network analysis, the SNP natural variations of *GWY10* between ZH11 and HX354 were queried in the haplotype network analysis tool (with R package) provided by RiceVarMap to draw a haplotype network plot. All natural variations are listed in **Supplementary Table 2**.

To examine the artificial selection of *OsBZIP47, GWY10*, and *CDKB2*, we performed the genetic diversity analysis. We retrieved the genetic variations within and flanking regions of *OsBZIP47*, *GWY10*, and *CDKB2* from the Rice SNP-Seek Database (https://snp-seek.irri.org). The retrieved variations were converted into the VCF format and performed their nucleotide diversity using VCFtools (0.1.16) for 203 rice accessions from mini-core collections.

**EMSA assays.** The *OsbZIP47* cDNA was amplified and fused in-frame into the pET32a vector (Invitrogen) and transformed into E.coli Rosetta (Novagen). OsbZIP47-His recombinant protein was purified using Glutathione Sepharose 4B beads (GE Healthcare). In brief, DNA probes (49 bp for *GWY10* from ZH11 and HX354, respectively) were synthesized (GENEray) (**Supplementary Table S4**) and labeled using a biotin label kit (Beyotime). OsbZIP47-His recombinant protein was incubated with the purified DNA probes at 4 °C in the EMSA binding buffer (Beyotime). DNA gel shift assays were performed using the Light Shift Chemiluminescent EMSA kit (Thermo Fisher Scientific). This experiment was repeated three times.

**RNA extraction and expression analysis.** Total RNA was extracted from the young panicle at various stages of development for differential expression analysis using TRizol RNA extraction kit (Invitrogen) following the manufacturing company’s protocol. Then reverse transcription was performed using M-MLV reverse transcriptase (Invitrogen) and 5X MasterMix (With genomic DNA removal kit) for cDNA synthesis. The expression of *GWY10* was performed by semi-quantitative RT-qPCR as well as by real-time PCR by using FastStart SYBR^®^ Green Master Mix and QuantStudio^TM^ 6 Flex system (Manufacturer’s standard protocol was applied. Rice ubiquitin gene (LOC_Os03g13170) was used as an internal control for the normalization of expression data. The primers used for expression analysis are listed in **Supplementary Table S4**. At least three biological replicates were used to determine the expression level.

**ChIP-qPCR assays.** Approximately 5g young leaves of mixed three independent transgenic lines of *35S:OsbZIP47-Flag* rice plants were fixed with 1% (v/v) formaldehyde under vacuum for 15min at 20–25 °C, and then homogenized in liquid nitrogen. Tissue fixation, nuclei extraction, and chromatin immunoprecipitation were conducted following established protocols. After isolation and lysing of nuclei, the chromatin complexes were isolated and ultrasonically fragmented into fragments with an average size of approximately 200bp for promoters. Immunoprecipitations were performed with anti-Flag antibodies (Sigma, F1804) overnight at 4°C. The precipitated DNA was recovered and dissolved in water and served as templates for RT-qPCR. Related primer sequences are given in **Supplementary Table S4**.

***In vitro* transient transactivation assays.** Approximately 2-Kb DNA promoter fragments of *GWY10* from ZH11 and HX354 were amplified, and then subcloned into a pUC19 vector containing the firefly luciferase reporter gene driven by the 35S minimal TATA box and 5×GAL4 binding elements, thus generating reporter plasmids containing specific promoters fused to luciferase. The full-length cDNA of *OsbZIP47* was amplified and fused to sequence encoding GAL4BD, thus generating the effector plasmid. Transient transactivation assays were performed using rice protoplasts as described previously. The Dual-Luciferase Reporter Assay System (Promega, E1960) was used to perform the luciferase activity assay, with the Renilla luciferase gene as an internal control. The activity of LUC and REN was assayed using the Dual-Luciferase Reporter Assay System (E1910, Promega). After transient transformation, protoplasts were incubated for 16h at 25 °C and the harvested protoplasts were lysed in 60 μl of Passive Lysis Buffer. 30 μl of this crude extract was assayed in 30 μl of Luciferase Assay Buffer mixed with Substrate, and the chemiluminescence was measured using the TECAN SPARK Microplate Reader with a 10-s delay. 30 μl of Stop and Glow™ Buffer mixed with the substrate was then added and a second chemiluminescence measurement was made with a 10-s delay. The ratio of LUC to REN activity was used as the relative promoter activity for each gene. Three biological replicates were assayed per construct. Relevant primer sequences are given in **Supplementary Table S4**.

**Split luciferase complementation assays.** The assay was performed as reported previously, we generated GWY10-cLUC, GW5-Cluc, GW5-nLUC, CDKB2-Nluc, CYCA2;3-Nluc and CYCA3;1-nLUC by fusing the respective CDS into pCAMBIA-split_cLUC and pCAMBIA-split_nLUC vectors. Sequencing corrected plasmids were transformed into Agrobacterium tumefaciens strain GV3101. Different overnight cultured GV3101 cells were mixed as indicated combinations to a final OD600 = 0.5. The mixtures were immediately centrifuged at 5,000g for 15 min at room temperature and resuspended in activation buffer (10 mM MES, pH 5.7, 150 μM acetosyringone and 10 mM MgCl_2_). After incubation for at least 2 h at room temperature with gentle agitation, the activated GV3101 cells were transformed into *N. benthamiana* leaves and expressed for another 2 days before the LUC activity measurement. For observing the LUC signals, 1 mM d-luciferin solution (E1602, Promega) was sprayed onto the leaves of *N. benthamiana* and they were kept in the dark for 5 min. The images were captured by a NightOWL II LB 983 imaging apparatus with CCD. All the primers used for cloning are listed in **Supplementary Table S4.**

**Pull-down assays.** To confirm the interaction between GWY10 and CDKB2, the CDSs of *GWY10* was inserted into pGEX-4T-1 vector (double digested by restriction enzymes EcoRI and XhoI) to construct GWY10-GST plasmid, while the CDSs of *CDKB2* was inserted into pET32a vector (double digested by restriction enzymes EcoRI and XhoI) to construct CDKB2-His plasmid. Plasmids were introduced into Escherichia coli BL21 (DE3) cells. The expression of corresponding proteins was induced with 0.5 mM IPTG at 28 °C for 12 h. Appropriate bacterial lysate combinations in GST-pulldown buffer (50 mM Tris-HCl pH 7.5, 1% Triton X-100, 5 mM MgCl_2_, 1 mM EDTA, 150 mM NaCl, 1 mM DTT and 1 mM PMSF) were incubated and pulled down by 20 μl GST beads (Glutathione Sepharose 4B, GE Healthcare) with gentle shaking at 4 °C for 4 h. The buffer was used to wash beads five times. The beads were then added to 50 µl 1X SDS-loading buffer and boiled for 5 min at 95 °C. The eluted proteins were separated by 10% SDS–PAGE gel and then transferred to membrane and detected with anti-GST (1:5,000, ABclone, AE001) or anti-His antibodies (1:10,000, ABclone, AE003). All the primers used for cloning are listed in **Supplementary Table S4.**
